# Phospholipid transfer protein: The link between lipid metabolism and inflammation in healthy pregnancy and pregnancy with cardiometabolic complications

**DOI:** 10.5937/jomb0-64876

**Published:** 2026-04-15

**Authors:** Marko Stanković, Jelena Munjaš, Minja Derikonjić, Aleksandra Željković, Jelena Vekić, Tamara Antonić, Daniela Ardalić, Milica Miljković-Trailović, Marija Šarić-Matutinović, Tamara Gojković, Snežana Jovičić, Željko Miković, Aleksandra Stefanović

**Affiliations:** 1 Gynaecology and Obstetrics Clinic Narodni Front, Belgrade; 2 University of Belgrade, Faculty of Pharmacy, Department of Medical Biochemistry, Belgrade; 3 University of Belgrade, Faculty of Medicine, Department of Gynaecology and Obstetrics, Belgrade

**Keywords:** gestational diabetes, hypertensive disorders of pregnancy, phospholipid transfer protein, pregnancy, resistin, fosfolipidni transfer protein, gestacijski dijabetes, hipertenzivni poremećaji u trudnoći, rezistin, trudnoća

## Abstract

**Background:**

Phospholipid transfer protein (PLTP) regulates high-density lipoprotein (HDL) remodelling by transferring cholesterol and phospholipids and affecting particle size and function. Pregnancy alters lipid metabolism, but the adaptation of PLTP to pregnancy remains poorly understood. This study investigated longitudinal changes in PLTP gene expression and concentration during healthy pregnancy and pregnancy with cardiometabolic complications, and their associations with maternal lipid metabolism and inflammatory status.

**Methods:**

We followed 84 healthy and 20 pregnant women who developed gestational diabetes mellitus and hypertensive disorders during pregnancy. PLTP gene expression, PLTP concentration, advanced lipid profile parameters, and inflammatory status were analysed throughout the trimesters.

## Abstract (sr)

**Uvod:**

Fosfolipidni transfer protein (PLTP) reguliše remodelovanje lipoproteina visoke gustine (HDL) prenosom holesterola i fosfolipida, menjajući njihovu veličinu i funkcionalnost. Trudnoća je praćena promenom u metabolizmu lipida, međutim, uloga PLTP-a tokom trudnoće još uvek nije u potpunosti razjašnjena. Ova studija je ispitivala longitudinalne promene ekspresije PLTP gena i koncentracije tokom zdrave trudnoće i trudnoće sa kardiometabolickim komplikacijama, kao i njihovu povezanost sa metabolizmom lipida i inflamatornim statusom majke.

**Metode:**

Praćeno je 84 zdravih trudnica i 20 trudnica kod kojih su se tokom trudnoće razvili gestacijski dijabetes mellitus ili hipertenzivni poremećaji. Ekspresija gena za PLTP, koncentracija PLTP-a, parametri proširenog lipidnog profila i inflamatorni status analizirani su tokom trimestra.

**Rezultati:**

Sličan tok promena ekspresije gena za PLTP i koncentracija PLTP-a uočen je u obe grupe, ali je ekspresija gena za PLTP u prvom trimestru bila značajno niža u grupi sa komplikacijama (p&lt;0,05). Analiza serumskih neholesterolskih sterola u HDL subfrakcijama (NCSHDL) i plazmatskih ceramida [ceramid C16:0 (Cer C16:0), ceramid C24:0 (Cer C24:0)] pokazala je značajno niže koncentracije dezmosterolaHDL (p&lt;0,05), kao i značajno više koncentracije C24:0 (p&lt;0,001) u grupi sa komplikacijama u prvom trimestru.

**Zaključak:**

Rezultati studije ukazuju na izmenjenu ekspresiju gena za PLTP, prošireni lipidni profil i inflamatorni status u prvom trimestru trudnoće sa kardiometaboličkim komplikacijama. Dobijeni rezultati naglašavaju značaj detaljnog ispitivanja metabolizma lipida i inflamatornih puteva u ranoj trudnoći u cilju unapređenja ishoda trudnoće.

## Introduction

Physiological adaptations in lipid metabolism during pregnancy are characterized by increased serum concentrations of total cholesterol (TC) and triglycerides (TG), accompanied by a moderate elevation in low-density lipoprotein cholesterol (LDL-C) levels. In contrast to dyslipidaemia in cardiometabolic diseases, lipid metabolism during healthy pregnancy exhibits a unique pattern: high-density lipoprotein cholesterol (HDL-C) levels rise by the end of the first trimester and then gradually decline as pregnancy progresses [1]. However, these trajectories of changes are not typically seen in cardiometabolic pregnancy complications (gestational diabetes and hypertensive disorders of pregnancy) [1]. Notably, aberrant or excessive alterations in maternal lipid metabolism have been strongly associated with adverse pregnancy outcomes [1][2]. Despite extensive research over recent decades, a comprehensive understanding of gestational lipid metabolism remains incomplete, particularly regarding alterations in the metabolism, structure, and functional properties of HDL particles.

A complex HDL composition that includes cholesterol, non-cholesterol sterols (NCSs), phospholipids, TG, and various proteins indicates diverse physiological functions. There is growing evidence that HDL's role extends beyond reverse cholesterol transport. This lipoprotein plays an important role in maintaining a healthy pregnancy by mitigating inflammation, improving vascular function, reducing oxidative stress, and potentially lowering the risk of cardiometabolic pregnancy complications [3][4]. Furthermore, HDL plays a supportive yet essential role in foetal development, especially during early stages when the foetus cannot meet its own cholesterol needs. HDL contributes significantly to the placental supply of cholesterol and other bioactive lipids, influencing multiple aspects of foetal growth and development [3]. Given the numerous functions of this lipoprotein, changes in its structure and function can be associated with an unfavourable pregnancy course and outcome. In confirming this, our previous study showed distinctive HDL protein composition in healthy and complicated pregnancies, which correlated with distinct HDL anti-inflammatory and antioxidative capacities [4].

The trajectory of changes in proteins involved in HDL lipid remodelling during pregnancy remains insufficiently characterized. Given the complexity of HDL metabolism during pregnancy, phospholipid transfer protein (PLTP) is an important molecule to investigate. PLTP facilitates HDL particle interconversion by promoting the formation of small (pre-β) HDL through ATP-binding cassette transporter ABCA1-mediated efflux of cellular cholesterol and phospholipids, as well as the generation of large, spherical mature HDL via the transfer of phospholipids and cholesterol from triglyceride-rich lipoproteins [5]. It is a key regulator of HDL metabolism, influencing HDL size, composition, and functional properties [6]. Studies in mouse models have shown that both PLTP deficiency and PLTP overexpression reduce cholesterol efflux capacity and alter HDL structure and function, ultimately diminishing HDL's antiatherogenic properties (6). Despite PLTP's central role in maintaining lipoprotein homeostasis, its behaviour during healthy pregnancy and in pregnancies complicated by cardiometabolic disorders remains unclear. Notably, previous studies have reported upregulation of PLTP gene expression in the oviductal epithelium of pregnant mice, suggesting a potential role for PLTP in regulating preimplantation embryo development in vivo [7]. Limited prior results suggest that PLTP contributes to HDL reassembly, which, in turn, affects the transport of cholesterol and phospholipids to the developing foetus [8][9].

Furthermore, PLTP has been shown to modulate inflammatory pathways, acting as a key determinant of the adaptive immune response, suggesting that during pregnancy, it can affect the balance between pro-inflammatory and anti-inflammatory cytokines [10]. No data, however, exists regarding the changes in *PLTP *gene expression and concentration during pregnancy with complications. The main hypothesis of this study is that *PLTP *gene expression and concentration are altered during pregnancy in women with cardiometabolic complications. To investigate the association of PLTP with inflammatory status and lipid metabolism throughout gestation, additional biochemical markers with established relevance were selected. Resistin was measured as a pro-inflammatory adipokine known to link systemic inflammation with dyslipidaemia, providing a focused assessment of inflammatory status [11]. To examine the contribution of PLTP to HDL remodelling and overall lipid metabolism, non-cholesterol sterols in HDL subfractions (NCSHDL) and two ceramide species [ceramide C16:0 (Cer C16:0) and ceramide C24:0 (Cer C24:0)] were selected, as these analytes are recognized as novel biomarkers of cardiovascular disease risk [12].

In this study, the longitudinal trajectory of changes in *PLTP* gene expression in peripheral blood mononuclear cells (PBMC) was analysed during healthy pregnancy and during pregnancy followed by cardiometabolic complications. Furthermore, potential associations between changes in *PLTP *gene expression and concentration with specific plasma lipids, inflammatory status, and the development of cardiometabolic pregnancy complications were investigated.

### Materials and methods

### Subjects

This retrospective case-control study represents a secondary analysis of a broader longitudinal research project (High-density lipoprotein MetabolOMe research to improve pregnancy outcome - HI-MOM). In the parent study, we enrolled 131 pregnant women without identified risk factors for pregnancy complications. Participants were recruited during their first routine gynecological examination at the Gynaecology and Obstetrics Clinic »Narodni Front« in Belgrade, Serbia. Exclusion criteria included pre-existing chronic diseases, acute infections at any point during follow-up, multiple gestations, molar pregnancies, and the use of medications known to affect lipid metabolism. Participants provided a detailed medical history, including information on systemic diseases, smoking status, alcohol consumption, vitamin supplementation, and family history of cardiovascular disease and diabetes mellitus. All women reported region-typical dietary habits and, although none were professionally engaged in sports, nearly all reported moderate physical activity. Standard prenatal vitamin and antioxidant supplementation was recommended to all participants.

All participants were followed prospectively throughout pregnancy until delivery, with clinical and full laboratory evaluations conducted during routine visits near the end of each trimester. The study protocol included anthropometric measurements for BMI calculation, comprehensive biochemical and haematological laboratory testing, and ultra-sonographic and Colour Doppler assessments for MUtA-PI calculation [13]. Mean arterial pressure (MAP) was determined following a standardized blood pressure measurement protocol [14].

After the follow-up period, the two study groups were defined based on pregnancy outcomes. The control group consisted of women with a healthy pregnancy outcome. In contrast, the group with complications included women who developed cardiometabolic pregnancy complications (gestational diabetes, gestational hypertension, or preeclampsia). To eliminate the potential influence of obesity on the pregnancy course and outcome, women with a pregestational BMI≥30 kg/m^2^ were excluded. In total, 5 women from the control group and 3 women from the complication group were excluded due to pregestational BMI≥30 kg/m^2^. Additionally, 19 women initially assigned to the group with complications were excluded because they developed pregnancy complications outside the cardiometabolic outcomes of interest. Ultimately, the control group comprised 84 pregnant women, and the complication group comprised 20 women who developed cardiometabolic complications [gestational diabetes (n=8); gestational hypertension (n=8); preeclampsia (n=2); combined gestational diabetes and gestational hypertension (n=2)]. The study objectives and protocol were explained in detail to the participants, and all provided written consent to participate. The local ethical committees approved the research after it had been planned in accordance with the Helsinki Declaration.

### Sample collection

This research was designed as a longitudinal study. After a nighttime fast (10 hours), venous blood from each subject's antecubital vein was taken toward the end of each trimester [first trimester (median: 13.2 gestational weeks), second trimester (median: 22.3 gestational weeks and third trimester (median: 32.9 gestational weeks)]. One serum sample tube and one EDTA sample tube were centrifuged at 1500 g for 10 minutes to obtain serum and plasma. They were aliquoted and stored at -80°C until analysis. One more EDTA sample tube was used for the isolation of peripheral blood mononuclear cells using Ficoll-Paque™ PLUS medium (GE Healthcare, Waukesha, Wisconsin, USA). Total RNA was extracted using TRIzol™ reagent (Ambion, Life Technologies, Grand Island, NY) following the manufacturer's protocol. The isolated RNA samples were stored at -80°C until further use in reverse transcription (RT) reactions.

### Methods

Glucose, high-sensitivity C-reactive protein (hs-CRP) concentration, and general lipid profile parameters [TG, TC, HDL-C] were measured using an automated biochemical analyser (AU 480, Beckman Coulter, USA) with commercially available manufacturer-provided reagent kits, in accordance with standardized laboratory protocols. Internal quality control procedures were applied daily, and calibration was performed according to the manufacturer's instructions to ensure analytical accuracy and precision. LDL-C concentrations were calculated using the Friedewald equation for samples with triglyceride concentrations within the validated analytical range.

PLTP concentration was determined using a commercially available sandwich enzyme-linked immunosorbent assay (ELISA) kit (Human PLTP ELISA Kit, Wuhan Fine Biotech Co., China). This assay is specifically designed for the quantitative detection of human PLTP, with no cross-reactivity with related proteins. The assay's analytical sensitivity was 1.875 ng/mL. The reported intra-assay and inter-assay coefficients of variation (CV) were 6.9% and 11.5%, respectively, indicating acceptable reproducibility.

Serum resistin concentrations were measured using a commercially available Human Resistin ELISA development kit (DuoSet DY1990, R&D Systems, Abingdon, UK/Minneapolis, USA), based on a quantitative sandwich immunoassay principle. According to the manufacturer's specifications, the intra-assay and inter-assay CVs were 6.0% and 10.0%, respectively, confirming good analytical precision. All samples were analysed according to the manufacturers' protocols, and measurements were performed in duplicate where applicable to minimize analytical variability.

RT and quantitative real-time PCR (qPCR) analyses were conducted on the 7500 Real-Time PCR System (Applied Biosystems, Foster City, CA, USA), and data were acquired in real-time mode using SDS software version 1.4.0.25. Complementary DNA (cDNA) synthesis was performed with the High-Capacity cDNA Reverse Transcription Kit (Applied Biosystems, Foster City, CA, USA). Quantitative realtime PCR was performed using 5'-nuclease TaqMan® gene expression assays (Applied Biosystems, Foster City, CA, USA) specific for human *PLTP *(Hs01067287_m1). Beta-actin (Hs99999903_m1) served as the endogenous control for normalization (housekeeping gene). According to the Minimum Information for Publication of Quantitative RealTime PCR Experiments Guidelines, the probe context sequence and amplicon context sequence for each TaqMan® gene expression assay are listed.

For the human PLTP (Hs01067287_m1) gene, the probe context sequence and amplicon context sequence are provided as follows:

Probe context sequence:

TGGGAGCATTGTCCTGCTGAGCCCA 

Amplicon context sequence:

AGCTGTTGCTGGTGGGGGACAAGGTG-CCCCACGACCTGGACATGCTGCTGAGGGC-CACCTACTTTGGGAGCATTGTCCTGCTGAGC-CCAGCAGTGATTGACTCCCCATTGAAGCTG-GAGCTGCGGGTCCTGGCCCCACCGCGCTGCAC-CATCA.

For the beta-actin (Hs99999903_m1) gene, the probe context sequence and amplicon context sequence are provided as follows:

Probe context sequence:

CCTTTGCCGATCCGCCGCCCGTCCA

Amplicon context sequence:

ACCGCCGAGACCGCGTCCGCCCCGCGAG-CACAGAGCCTCGCCTTTGCCGATCCGCCGC-CCGTCCACACCCGCCGCCAGCTCACCATGGAT-GATGATATCGCCGCGCTCGTCGTCGACAACG-GCTCCGGCATGTGCAAGGCCGGCTTCGCGGGC-GACGATGCCCCCCGGGCCGTCTTCCCCTCCATC-GTGGGGCGCCCCAGGCACCAGGGCGTGATG-GTGGGCAT.

Following RT, all cDNA samples were pooled, and five serial dilutions of the pooled material were prepared to generate standard curves for both the target and housekeeping genes. Each 15 μL PCR reaction contained 5 μL of cDNA. PCR amplification was performed using a thermal cycler with the following program: an initial 15-minute activation step at 95°C to trigger the HOT FIREPol® DNA Polymerase (Solis BioDyne, Tartu, Estonia), followed by 40 cycles consisting of a 15-second denaturation at 95°C and a 60-second annealing/extension phase at 60°C. The intra- and inter-assay coefficients of variation for the human *PLTP *gene were 0.038% and 0.16%, respectively. In comparison, those for the human β-actin gene were 0.08% and 0.14%, respectively. Relative gene expression was determined using the relative standard curve method. A standard curve was generated for both the target gene and an endogenous control (or reference) gene using a common sample dilution series to determine amplification efficiency. The Cq values for experimental samples were used with these standard curves to extrapolate target and reference gene quantities, which were then normalized and presented as a ratio of the target gene mRNA to the housekeeping gene mRNA.

Serum cholesterol precursors ((NCSs) (desmosterol, 7-dehydrocholesterol, and lathosterol) and cholesterol absorption markers (campesterol and β-sitosterol), in HDL subfraction (NCSHDL), were determined by liquid chromatography-tandem mass spectrometry (LC-MS/MS) as previously described in detail [15]. Desmosterol, 7-dehydrocholesterol, lathosterol, campesterol, β-sitosterol, and d_6_-cholesterol (internal standard) were quantified using HPLC-grade standards (Sigma-Aldrich, USA). ApoB-depleted serum was obtained by mixing 200 μL of serum with 500 μL of phosphotungstate/MgCl_2_ precipitation reagent (BioSystems, Spain), followed by incubation (10 min, RT) and centrifugation (6000 rpm, 10 min). A 650 μL aliquot of the supernatant (HDL fraction) was transferred to glass tubes precoated with d_6_-cholesterol (50 μL, 1 mg/mL), followed by the addition of 1 mL 2% ethanolic KOH and incubation at 45°C for 30 min for sterol ester hydrolysis. After cooling to ambient temperature, each sample was supplemented with 500 μL of HPLC-grade water. Subsequently, 2 mL of n-hexane was added, and the mixture was vigorously vortex-mixed for 30 s. Phase separation was achieved by centrifugation at 1500xg for 5 min, after which the upper organic phase was carefully transferred to a clean glass tube. This liquid-liquid extraction with n-hexane was repeated twice more, and all collected organic phases were pooled. To remove residual KOH, 4 mL of HPLC-grade water was added to the combined organic extracts, followed by centrifugation under the same conditions (5 min, 1500xg). The purified organic phase was then collected, transferred into a clean glass tube, evaporated to dryness under a gentle stream of nitrogen, and reconstituted in 20 μL of HPLC-grade methanol. A 10-μL aliquot of the final methanolic extract was injected onto the chromatographic system. Liquid chromatographic analysis was performed using an Agilent 1290 UHPLC system equipped with a Poroshell 120 EC column (2.5 pm, 4.6x150 mm; Agilent Technologies, Santa Clara, CA, USA). Chromatographic separation was achieved under isocratic conditions using a mobile phase composed of acetonitrile, methanol, and water containing 0.1% formic acid (80:18:2, v/v) at a flow rate of 0.6 mL/min and a column temperature of 30°C, with a total run time of 45 min. Detection and quantification were performed using an Agilent 6420 triple quadrupole mass spectrometer with APCI in positive-ion mode. Source parameters included: gas temp 325°C, vaporizer 250°C, gas flow 5 L/min, nebulizer 30 psi, and capillary voltage +2000 V. Intra- and inter-assay CVs were 2.5-13.6%, with analyte recoveries of 85.3-95.8%.

Quantification of ceramides [ceramide C16:0 (Cer C16:0), ceramide C24:0 (Cer C24:0] was done by the HPLC-MS/MS method, previously described in detail [16]. HPLC-grade analytical standards (Avanti Polar Lipids, Birmingham, AL, USA) were used to quantify ceramides. A 50 μL aliquot of plasma was transferred into glass vials pre-coated with internal standards. Subsequently, 2 mL of extraction solvent (methanol:chloroform, 2:1, containing 0.1 % trifluoroacetic acid) was added to each vial, followed by vigorous vortex mixing for 30 s. Thereafter, 0.67 mL of chloroform was introduced, and the samples were vortexed again for 30 s. An additional 1.15 mL of HPLC-grade water was then added to achieve a final methanol:chloroform:water ratio of 1:1:0.9. After vortex mixing for 30 s, the samples were centrifuged at 2,000xg for 20 min, resulting in the formation of a distinct protein interface. The lower (chloroform) phase was carefully collected, transferred to clean tubes, evaporated to dryness, and reconstituted in 30 μL of HPLC-grade methanol. The reconstituted extracts were vortexed for 30 s and centrifuged at 1,500xg for 10 min before injection into the chromatograph. Chromatographic separation of sphingolipids was performed using a Zorbax Eclipse Plus C8 column (4.6x150 mm, 5 μm; Agilent Technologies, Santa Clara, CA, USA) with a gradient elution of solvent A (1 mM ammonium formate in methanol with 0.2% formic acid) and solvent B (2 mM ammonium formate in water with 0.2% formic acid). The chromatographic run commenced with a mobile phase composition of 80% solvent A and 20% solvent B. A linear gradient was applied to increase solvent A to 90% at 10.6 minutes, and this composition was maintained for an additional 6 minutes. The gradient was then continued to reach 99% solvent A at 66 minutes, followed by a return to the initial condition of 80% solvent A at 68 minutes. This final composition was held for an additional 7 minutes to complete the run. The flow rate was set to 0.5 mL/min, and the injected sample volume was 15 μL. Quantification was conducted in multiple reaction monitoring mode on an Agilent 6420 triple quadrupole mass spectrometer equipped with an electrospray ionization source. Source parameters were set as follows: gas temperature at 340°C, vaporizer temperature at 250°C, nebulizer pressure at 20 psi, drying gas flow rate at 12 L/min, positive-ion capillary voltage at +4500 V, and negative-ion capillary voltage at -4000 V.

### Statistical analysis

Before statistical testing, the distributional properties of all continuous variables were examined using the Kolmogorov-Smirnov test to assess deviations from normality. This step enabled the appropriate selection of parametric or non-parametric statistical procedures. Variables with Gaussian distributions were summarized as arithmetic means with corresponding standard deviations (SD), reflecting central tendency and dispersion. Variables exhibiting log-normal distribution were logarithmically transformed and presented as geometric means with 95% confidence intervals (CI), thereby providing a more accurate estimate of central tendency for right-skewed data. For variables that remained non-normally distributed even after logarithmic transformation, results were expressed as medians with interquartile ranges (IQR; 25th-75th percentile). Categorical variables were summarized using absolute counts and relative frequencies (percentages).

Longitudinal comparisons of continuous variables across time points were performed using repeated-measures analysis of variance (ANOVA) for normally and log-normally distributed data, enabling assessment of within-subject effects over time. When overall significance was detected, Bonferroni post hoc correction was applied to control for type I error due to multiple pairwise comparisons. For variables that did not meet parametric assumptions, the Friedman test (non-parametric repeated-measures ANOVA) was used, followed by Wilcoxon signed-rank tests for post hoc pairwise comparisons.

Between-group comparisons were conducted according to data distribution. For normally and log-normally distributed variables, differences between two independent groups were analysed using Student's t-test. For variables with asymmetric (non-normal) distributions, the Mann-Whitney U test was applied. Associations between categorical variables were examined using the Chi-square test.

Correlation analyses were performed using Spearman's rank correlation coefficient, given its suitability for non-parametric data and for detecting monotonic relationships between variables.

To further explore potential associations between the investigated parameters and the occurrence of pregnancy complications, both univariate and multivariate binary logistic regression analyses were conducted. In these models, the absence of pregnancy complications (control group) was coded as the reference category (0). In contrast, the presence of complications was coded as the outcome category (1). Univariate logistic regression analysis was first performed to identify individual predictors significantly associated with the outcome. Variables demonstrating statistical significance in univariate analysis were subsequently entered into multivariate logistic regression models to determine independent predictors after adjustment for potential confounding factors. Results were expressed as odds ratios (OR) with corresponding 95% confidence intervals.

A two-tailed p-value <0.05 was considered indicative of statistical significance. All statistical analyses were performed using PASW Statistics software, version 18 (IBM Corp., Armonk, NY, USA).

## Results

Baseline characteristics of the study participants are presented in Table 1. Pregnant women in the group with complications were significantly older. They had significantly higher pregestational body mass index (BMI) compared with pregnant women in the control group (p<0.05). The two investigated groups did not differ in smoking habits before pregnancy, pregestational prenatal vitamin supplementation, and pregnancy weight gain (Table 1).

**Table 1:** General characteristics of study groups. Data are presented as mean ± standard deviation and compared by the Student t-test.<br># Data are presented as relative frequencies and compared by the Chi square test.<br>* Data are presented as median (interquartile range) and compared by the Mann-Whitney U-test.

	Control group	Group with complications	P
N	84	20	
Age (years)	31.6±5.42	35.2±5.91	<0.05
Pregestational BMI (kg/m^2^)	21.7±2.89	25.9±4.43	<0.001
Smoking before pregnancy (%)#	27.4	30	0.519
Pregestational prenatal vitamin<br>supplementation (%)#	35.7	50	0.129
Pregnancy weight gain (kg)*	17.5 (11.5-22.5)	15.0 (9.5-2.5)	0.092
Gestational diabetes (N)	-	8	
Gestational hypertension (N)	-	8	
Preeclampsia (N)	-	2	
Gestational diabetes and gestational<br>hypertension (N)	-	2	

Table 2 presents longitudinal changes in mean pulsatility index (Mean UtA-PI), MAP, glucose, and lipid profile parameters across trimesters in the study groups. In both investigated groups, we observed a significant decrease in Mean UtA-PI throughout pregnancy (*p*<0.001). In the control group, mean arterial pressure (MAP) significantly decreased in the second and third trimesters compared with the first (*p*<0.001). The same trend of changes was observed in the group with complications, too (*p*<0.05). A significant decrease in glucose concentrations in the second and third trimesters was observed in the control group (*p*<0.05), whereas there was no difference in the group with complications (*p*=0.379). TC, TG, and LDL-C concentrations increased significantly during pregnancy in both investigated groups. A significant increase in HDL-C concentration was observed in the second trimester compared with the first (*p*<0.001), followed by a significant decrease in the third trimester (*p*<0.05) in the control group. In contrast, no differences in HDL-C concentration were detected across trimesters in the group with complications.

**Table 2:** Changes in Mean UtA-PI, MAP glucose, and general lipid profile parameters concentrations in study groups across trimesters of pregnancy. Data are presented as mean ± standard deviation.<br>† Data are presented as geometric mean (95th CI).<br>*p* - Student t-test.<br>*p1* - ANOVA repeated measures for control group; p2 - ANOVA repeated measures for group with complications. Pairwise comparison: a mean difference significantly different from the 1st trimester; b mean difference significantly different from the 2nd trimester; * *p*<0.001 (Bonferroni corrected); # p<0.05 (Bonferroni corrected).

	1^st^ trimester			2^nd^ trimester			3^rd^ trimester			*p1*	*p2*
	Control<br>group<br>(N = 84)	Group with<br>complications<br>(N=20)	p	Control<br>group<br>(N = 84)	Group with<br>complications<br>(N = 20)	p	Control<br>group<br>(N=84)	Group with<br>complications<br>(N = 20)	*p*		
Mean<br>UtA-PI	1.7±0.52	1.4±0.43	<0.05	0.8±0.24^a*^	0.9±0.25^a*^	0.632	0.7±0.17^a*b*^	0.7±0.19^a* b#^	0.833	<0.001	<0.001
MAP	87.1±9.17	95.9±9.88	<0.001	81.5±9.52^a*^	91.6±9.82^a#^	<0.001	82.9±8.75^a*^	87.2±13.25^a#^	0,074	<0.001	<0.05
Glucose,<br>mmol/L	4.6±0.40	5.0±0.79	<0.05	4.4±0.47^a#^	4.7±0.57	0.051	4.5±0.46^a#^	4.9±0.87	<0.05	<0.05	0.379
TC, mmol/L	5.3±0.97	5.2±0.87	0.472	6.7±1.34^a*^	6.6±1.42^a*^	0.474	7.3±1.44^a*b^	6.4±1.44^a*^	<0.05	<0.001	<0.001
TG,<br>mmol/L†	1.23<br>(1.13-1.35)	1.51<br>(1.32-1.72)	<0.05	1.82^a*^<br>(1.66-1.99)	2.10^a*^<br>(1.86-2.36)	<0.05	2.28^a*,b*^<br>(2.06-2.52)	2.60^a*, b#^<br>(2.35 - 2.88)	0.258	<0.001	<0.001
HDL-C,<br>mmol/L	1.7±0,31	1.7±0.34	0.316	1.9±0.32^a*^	1.8±0.38	0.250	1.8±0.29^a#,b#^	1.7±0.35	0.420	<0.001	0.254
LDL-C,<br>mmol/L	3.0±0.86	2.8±0.69	0.177	4.1±1.21^a*^	3.5±1.01^a#^	0.054	4.2±1.22^a*,b#^	3.3±1.25	<0.05	<0.001	<0.05

Mean UtA-PI was significantly lower (*p*<0.05) while MAP, glucose, and TG concentrations were significantly higher in the group with complications compared with the control group in the first trimester. MAP and TG concentrations remained significantly higher in the group with second-trimester complications (Table 2). In the third trimester, glucose concentration was significantly higher (*p*<0.05) while TC and LDL-C concentrations were significantly lower in the group with complications than in the control group.

Next, we examined longitudinal alterations in *PLTP *gene expression, PLTP, hsCRP, and resistin concentrations in study groups (Table 3). In both investigated groups, PLTP gene expression increased significantly in the second trimester, followed by a significant decrease in the third trimester (*p*<0.001). In the control group, PLTP concentration increased significantly in the second trimester (*p*<0.001). It remained elevated until the end of pregnancy. In the group with complications, a significant increase in PLTP concentration was observed in the third trimester (*p*<0.05). We did not find significant changes in hsCRP concentrations across trimesters in either group (Table 2).

**Table 3:** Changes in PLTP gene expression, PLTP concentration and inflammatory markers in study groups across trimesters of pregnancy. Data are presented as median (interquartile range).<br>*p* - Mann-Whitney U-test; *p1* - Friedman test (Non-parametric ANOVA repeated measures) for control group; *p2 *- Friedman test (Non-parametric ANOVA repeated measures) for group with complications; Pairwise comparison: ^a^significantly different from the 1^st^ trimester; ^b^significantly different from the 2^nd^ trimester; *p<0.001 (Wilcoxon signed-rank test);^ #^*p*<0.05 (Wilcoxon signed-rank test).

	1st trimester			2nd trimester			3rd trimester			*p1*	*p2*
	Control<br>group<br>(N = 84)	Group with<br>complications<br>(N=20)	p	Control<br>group<br>(N = 84)	Group with<br>complications<br>(N=20)	*p*	Control<br>group<br>(N = 84)	Group with<br>complications<br>(N=20)	*p*		
PLTP<br>mRNA	0.92<br>(0.72-1.15)	0.7<br>(0.59-0.95)	<0.05	1.12^a#^<br>(0.74-1.42)	1.02^a#^<br>(0.64-1.52)	0.739	0.69^a*,b*^<br>(0.34-1.01)	0.58^a#,b*^<br>(0.29-0.76)	0.138	<0.001	<0.001
PLTP<br>mg/mL	712.8<br>(520.5-988.1)	647.4<br>(394.8-773.3)	0.521	1024.9^a#^<br>(630.9-1827.7)	863.9<br>(620-1966.3)	0.194	1180^a#^<br>(1082.8-1442.3)	1349.8^a#^<br>(887.5-1667.6)	0.957	<0.001	<0.05
hsCRP<br>mg/L	4.0<br>(2.4-7.4)	3.9<br>(1.97-12.25)	0.720	4.5<br>(2.3-8.7)	3.6<br>(1.8-9.8)	0.773	4.1<br>(2.9-7.0)	2.5<br>(1.6-9.8)	0.354	0.180	0.386
Resistin,<br>pg/L	21.8<br>(15.8-30.8)	30.4<br>(19.9-38.9)	<0.05	52.6^a*^<br>(26.6-92.4)	60.9^a#^<br>(29.4-101.9)	0.458	44.5 a*<br>(33.2-63.8)	41.4<br>(31.4-71.5)	0.671	<0.001	<0.05

In contrast, resistin concentrations significantly increased in both groups in the second trimester. PLTP gene expression was significantly lower (*p*<0.05). At the same time, resistin concentrations were significantly higher (*p*<0.05) in the group with first-trimester complications. There were no significant differences in other investigated parameters between the control group and the group with complications across trimesters of pregnancy (Table 3).

To gain deeper insight into lipid profile changes, we followed the longitudinal trajectories of NCSHDL and plasma ceramide concentrations (Cer C16:0 and Cer C24:0) across study groups (Table 4). In both investigated groups, we noticed a significant increase in desmosterol HDL concentrations [in the third trimester in the control group and in the second trimester in the group with complications (*p*<0.001 vs <0.05)]. Lathosterol HDL concentration in the control group significantly increased in the second trimester (*p*<0.05). It remained higher in the third trimester (*p* < 0.05). Cer C16:0 and Cer C24:0 concentrations in the control group showed a significant increase in the second trimester compared with the first and then a significant increase in the third trimester compared with the second (*p*<0.001).

**Table 4:** Changes in NCSHDL and ceramides in study groups across trimesters of pregnancy. Data are presented as median (interquartile range).<br>*p* - Mann-Whitney U-test; *p1* - Friedman test (Non-parametric ANOVA repeated measures) for control group; *p2* - Friedman test (Non-parametric ANOVA repeated measures) for group with complications; Pairwise comparison: ^a^significantly different from the 1^st^ trimester; ^b^ignificantly different from the 2^nd^ trimester; *p<0.001 (Wilcoxon signed-rank test); #*p*<0.05 (Wilcoxon signed-rank test).

	1st trimester			2nd trimester			3rd trimester			p1	p2
	Control<br>group<br>(N=84)	Group with<br>complications<br>(N=20)	P	Control<br>group<br>(N=84)	Group with<br>complications<br>(N=20)	p	Control<br>group<br>(N=84)	Group with<br>complications<br>(N=20)	p		
Desmosterol<br>HDL, mmol/L	0.26<br>(0.20-0.31)	0.19<br>(0.15-0.23)	<0.05	0.27(0.23-0.35)	0.32^a#^<br>(0.24-0.41)	0.077	0.32^a*b#^(0.24-0.42)	0.28^a*^<br>(0.23-0.40)	0.280	<0.001	<0.05
7-DHCHDL,<br>mmol/L	0.39<br>(0.33-0.47)	0.38<br>(0.30-0.51)	0.961	0.40(0.35-0.51)	0.46<br>(0.37-0.64)	0.083	0.41<br>(0.34-0.49)	0.39<br>(0.29-0.48)	0.463	0.211	0.819
Lathosterol<br>HDL, mmol/L	1.06<br>(0.71-1.53)	1.17<br>(0.89-2.14)	0.181	1.34 a*<br>(0.91-1.72)	1.57<br>(0.95-2.05)	0.297	1.34^a*^<br>(0.96-1.93)	1.29<br>(1.04-2.16)	0.862	<0.001	0.387
Campesterol<br>HDL, mmol/L	0.54<br>(0.36-0.72)	0.40<br>(0.28-0.62)	0.144	0.52<br>(0.36-0.72)	0.39<br>(0.28-0.59)	0.091	0.47<br>(0.31-0.64)	0.37<br>(0.28-0.51)	0.181	0.272	0.549
b-Sitosterol<br>HDL, mmol/L	5.6<br>(4.16-8.63)	4.74<br>(3.32-7.14)	0.240	5.7<br>(4.37-9.09)	4.74<br>(2.78-5.99)	<0.05	5.2<br>(4.23-8.94)	4.81<br>(3.85-5.97)	0.163	0.05	0.678
Cer C16:0,<br>mmol/L	0.36<br>(0.30-0.45)	0.34<br>(0.19-0.39)	0.448	0.38^a*^<br>(0.29-0.49)	0.38<br>(0.25-0.48)	0.840	0.47^a*,b*^<br>(0.35-0.61)	0.39<br>(0.28-0.58)	0.306	<0.001	0.05
Cer C24:0,<br>mmol/L	1.18<br>(0.57-1.59)	1.94<br>(1.68-2.34)	<0.001	1.9^a*^<br>(1.16-2.57)	2.09<br>(1.15-3.28)	0.783	2.73»^a*,b*^<br>(1.54-3.89)	2.27<br>(1.45-3.84)	0.683	<0.001	0.472

In contrast, except for desmosterol HDL, we found no differences in any other lipid profile parameters in the group with complications across trimesters of pregnancy (Table 4). In the group that experienced complications, their desmosterol HDL concentrations were significantly lower (*p*<0.05), while Cer C24:0 concentrations were significantly higher (*p*<0.001) in the first trimester compared with the control group. In the second trimester, only β-sitosterol HDL was significantly lower in the group with complications (*p*<0.05). No additional significant differences in the investigated advanced lipid profile parameters were detected between the control group and the group with complications throughout pregnancy.

Additionally, all first-trimester parameters that significantly differed between the groups were entered into multivariate binary regression models to evaluate whether the observed differences could be attributed to maternal age and pregestational BMI. Spearman's correlation test was used for correlation analysis. We found statistically significant negative correlations between PLTP and resistin concentrations in the control group in the first (p = 0.231; *p*<0.05), second (p=-0.415; *p*<0.001), and third trimester (p=-0.345; *p*<0.05). *PLTP *concentration was in significant positive correlation with desmosterolHDL (p=0.227; *p*<0.05) and in significant positive correlation with β-sitosterolHDL (p = 0.226; *p*<0.05) in the control group in the first trimester. Our results indicate a significant positive correlation between PLTP gene expression and Cer C24:0 concentrations in the control group in the first trimester. Correlation analysis did not reveal a significant association between PLTP concentration and inflammatory markers in the group with complications (data not shown). We found a significant negative correlation between *PLTP *gene expression and TG and desmosterolHDL concentrations in the first trimester in the group with complications (p=-0.592; *p*<0.001 vs p=-0.496; *p*<0.05).

Next, univariate binary logistic regression analysis was applied to evaluate the ability of the investigated parameters to predict the development of pregnancy complications (Table 5). Results of univariate analysis highlighted age, pregestational BMI, MAP, mean UtA-PI, glucose, TG, PLTP gene expression, resistin, desmosterolHDL, and Cer C24:0 levels in the first trimester as potential markers for identifying pregnancy complications.

**Table 5:** U nivariate binary logistic regression analysis for associations between the examined parameters and pregnancy complications development. OR - Odds ratio; p - Binary logistic regression test.

Parameter	OR	95%CI	P
Age, years	1.131	1.029-1.242	<0.05
Pregestational BMI, kg/m^2^	1.358	1.164-1.583	<0.05
MAP	1.102	1.040-1.169	<0.05
Mean UtA-PI	0.316	0.108-0.927	<0.05
Pregestational prenatal vitamin<br>supplementation	0.667	0.190-2.338	0.526
Smoking before pregnancy	1.208	0.413-3.532	0.730
Glucose, mmol/L	3.768	1.451-9.785	<0.05
TG, mmol/L	2.857	1.125-7.252	<0.05
*PLTP *mRNA	0.103	0.013-0.794	<0.05
Resistin, pg/L	1.040	1.004-1.076	<0.05
DesmosterolHDL, mmol/L	0.001	0.001-0.064	<0.05
Cer C24:0, mmol/L	2.970	1.598-5.521	<0.05

Further, a logistic regression model was created to examine the potential independent association of the investigated parameters of interest with the onset of cardiometabolic pregnancy complications (Table 6). The model incorporated traditional risk factors for the development of cardiometabolic pregnancy complications, which were significant predictors (*p*<0.1) in univariate analysis (age, pregestational BMI, MAP, Mean UtA-PI, glucose, TG, and resistin), and added PLTP gene expression as a parameter of interest for further analysis. The observed predictive capacities of age, MAP, Mean UtA-PI, glucose, TG, and resistin concentrations were lost after accounting for the joint effects of all significant predictors in the model. Still, higher pregestational BMI and lower PLTP gene expression remained strongly associated with the development of pregnancy complications, regardless of other variables. Those parameters have shown significant, independent associations with the development of pregnancy complications (Nagelkerke R Square 0.551).

**Table 6:** Multivariate binary regression analysis for independent associations between significant parameters for pregnancy complications development. OR - Odds ratio; p - Binary logistic regression test<br>Nagelkerke R Square (percentage of variation explained by the predictors)

Parameter	OR	95%CI	P1
Age, years	1.144	1.00-1.306	0.051
Pregestational BMI, kg/m^2^	1.301	1.016-1.665	**<0.05**
MAP	1.055	0.972-1.145	0.198
Mean UtA-PI	0.345	0.080-1.486	0.118
Glucose, mmol/L	2.383	0.654-8.685	0.188
TG, mmol/L	3.345	0.736-15.192	0.118
Resistin, pg/mL	1.059	0.987-1.136	0.112
*PLTP*, mRNA	0.007	0.001-0.243	**<0.05**
Model Summary: Nagelkerke R Square 0.579			

## Discussion

In this study, gestational changes in *PLTP* gene expression and circulating PLTP concentrations were longitudinally evaluated in women with favourable pregnancy outcomes and in those who subsequently developed cardiometabolic complications, including gestational diabetes mellitus and hypertensive disorders of pregnancy. Possible associations of *PLTP *gene expression and concentration with maternal inflammatory status and the advanced lipid profile during pregnancy were investigated. The results indicate that the trajectories of change in *PLTP *gene expression and PLTP concentrations were similar in healthy pregnancies and those complicated by cardiometabolic disorders. However, first-trimester *PLTP *gene expression was significantly higher in the group with complications. In the initial trimester, the cardiometabolic complication group also demonstrated lower desmosterolHDL, alongside higher Cer C24:0 and resistin concentrations. Moreover, *PLTP *gene expression in the initial trimester was identified as being independently associated with the development of cardiometabolic pregnancy complications [16].

Advanced maternal age and elevated prepregnancy BMI are well-established independent and interacting risk factors for cardiometabolic pregnancy complications [17][18]. Our findings align with this evidence, since women who developed cardiometabolic complications were significantly older and had higher pre-gestational BMI than controls (Table 1). Although participants with prepregnancy obesity were excluded and gestational weight gain did not differ between groups (Table 1), the results indicate that even pre-gestational overweight, particularly when combined with advanced maternal age, heightens the risk of cardiometabolic pregnancy complications. These observations underline the importance of maternal metabolic health before conception in ensuring favourable pregnancy outcomes.

Mean UtA-PI decreased across gestation in both groups (Table 2), consistent with recently established gestational-age-specific reference ranges [19]. Notably, Mean UtA-PI was significantly lower in the group with first-trimester complications (Table 2). However, evidence regarding its predictive value remains inconsistent, and current consensus holds that Mean UtA-PI should be interpreted alongside maternal risk factors, laboratory evaluation, and foetal measurements when assessing risk of pregnancy complications [20].

During physiological pregnancy, maternal lipid metabolism undergoes characteristic adaptations, including progressive increases in TG and moderate elevations in TC, LDL-C, and HDL-C [1][21]. These physiological changes support fatal growth and maternal energy needs. They are not considered atherogenic, partly because lipid levels return to baseline postpartum [21][22]. Higher HDL-C during pregnancy is increasingly recognized as an atheroprotective feature of this adaptation [23]. Our findings reflect these expected lipid trajectories in both healthy and complicated pregnancies (Table 2). Notably, women with cardiometabolic complications demonstrated markedly elevated triglyceride concentrations during both the first and second trimesters, as well as a lack of a typical increase in HDL-C levels (Table 2). These findings align with previously reported characteristics of cardiometabolic pregnancy dyslipidemia, which is marked by pronounced hypertriglyceridemia and an absence of HDL-C elevation [24].

To gain deeper insight into HDL remodelling during pregnancy, we investigated longitudinal changes in maternal PLTP gene expression in PBMCs. PLTP is expressed in various tissues, including the placenta, but a growing body of evidence suggests that PBMCs are acceptable surrogate models for investigation [25]. Our study results indicate the same trajectory of changes in *PLTP *gene expression in both investigated groups (Table 3). PLTP gene expression was significantly increased in the second trimester and subsequently decreased in the third trimester (Table 3). Interestingly, PLTP concentrations in both investigated groups increased throughout pregnancy (Table 3). Furthermore, significantly lower *PLTP *gene expression in the first trimester was observed in the group with complications. To our knowledge, no previous studies longitudinally monitored this protein during pregnancy. Bringing to mind the PLTP's crucial role in lipoprotein metabolism, an increase in *PLTP *gene expression and PLTP concentration in midpregnancy reveals maternal metabolic adaptations to gestational dyslipidaemia. Our advanced lipid profile analysis showed significant differences between the investigated groups (Table 4). In both groups, we found that only desmosterol HDL concentration significantly increases during the course of pregnancy (Table 4). In the control group alone, we observed a significant increase in lathosterol HDL, Cer C16:0, and Cer C24:0 concentrations throughout the course of pregnancy. These results are in agreement with recently published research, which emphasized that longitudinal lipidomic profiling during pregnancy can indicate the cardiometabolic risk [26]. In light of our results, we speculate that the absence of an increase in cholesterol precursors' concentrations in the HDL subfraction and in plasma Cer C16:0 and Cer C24:0 concentrations during pregnancy is a hallmark of cardiometabolic pregnancy complications. Advanced lipid profiling in our study also highlighted the importance of metabolic status in the first trimester of pregnancy for pregnancy outcome. We found significantly higher desmosterolHDL and significantly lower Cer C24:0 concentrations in the control group compared with the group with complications (Table 4). Higher desmosterolHDL concentration in the HDL subfraction in the initial trimester and an increase in the other precursor, lathosterol HDL, can be indicators of higher HDL-C concentration in healthy pregnancy. These results confirmed the previous statements that understanding the molecular details of HDL composition and function during healthy pregnancy requires identifying any potential alterations associated with adverse outcomes [27]. Ceramides have been widely studied for their roles in cellular signalling and their potential as biomarkers [28]. Their involvement in pregnancy, however, remains insufficiently understood. Although first-trimester ceramide levels have been proposed as promising biomarkers for cardiometabolic pregnancy complications, data are still limited and inconsistent [29]. Elevated first-trimester ceramide levels have been suggested as early predictors of gestational diabetes [30][31], and growing evidence links ceramides to the pathogenesis of preeclampsia [32][33]. The higher Cer C24:0 levels observed in our group with complications support these earlier reports. Given their involvement in inflammation, apoptosis, and fibrosis, ceramides are increasingly recognized as independent risk predictors and potential therapeutic targets [34]. However, current studies on first-trimester ceramide concentrations and pregnancy complications face a lot of limitations. Retrospective design with small cohorts, differences in measurement techniques, and a lack of standardized reference ranges reduce consistency across studies. To consider ceramides for clinical use, a better understanding of their role in both healthy pregnancies and cardiometabolic pregnancy complications is necessary. Our study aimed to find possible associations between *PLTP *gene expression and concentration with advanced lipid profile markers. Our results indicated the importance of first-trimester status, and correlation analysis showed that PLTP concentration was significantly positively correlated with desmosterol HDL and β-sitosterol HDL in the control group. Furthermore, a positive correlation between *PLTP *gene expression and Cer C24:0 concentrations was observed in the control group during the first trimester. On the other side, in the group with complications, we found a significant negative correlation between *PLTP* gene expression and TG and desmosterol HDL concentrations in the first trimester. Thus, our results strongly highlighted the connections between PLTP and advanced lipid profiles in both healthy and cardiometabolic-complication pregnancies. We found a direct connection between PLTP concentration and cholesterol precursors within HDL particles. We confirmed the crucial role of PLTP in HDL shaping and remodelling in healthy pregnancy. However, this association was not observed in cardiometabolic pregnancy complications. According to our results, the metabolic adaptation in pregnancy with cardiometabolic complications is characterized by lower PLTP gene expression, which is strongly associated with higher TG concentrations and lower cholesterol precursor content in HDL particles.

Beyond its influence on plasma lipoprotein remodelling, PLTP also plays a key role in modulating the immune response, with both pro-inflammatory and anti-inflammatory properties [10][35]. Previous studies have shown positive associations between PLTP concentration and activity and various inflammatory markers in cardiovascular disease and type 2 diabetes mellitus [36][37]. Furthermore, it has been shown that PLTP's implication in immune response extends to its role in the pathogenesis of metabolic syndrome and atherosclerosis. Elevated PLTP activity has been associated with increased production of very low-density lipoprotein and alterations in HDL composition, both of which contribute to atherosclerosis development. The inflammatory milieu in these conditions may further modulate PLTP activity, creating a vicious circle that intensifies dyslipidaemia and vascular inflammation [38]. During a healthy pregnancy, the maternal immune system undergoes dynamic but precisely controlled changes. Early gestation and placental development occur in a pro-inflammatory milieu, whereas subsequent phases require an anti-inflammatory environment to facilitate maternal tolerance of the foetus [39]. In this study, we observed a significant increase in resistin concentrations during the second trimester in both study groups.

Furthermore, resistin levels in the first trimester were significantly higher in the group that developed pregnancy complications (Table 3). These findings are consistent with previous research, indicating that resistin may modulate immune responses, contribute to insulin resistance, influence angiogenic processes, and impact pregnancy outcomes, thereby suggesting that elevated resistin levels could be associated with the pathogenesis of pregnancy complications [11][40]. Notably, no significant differences in hsCRP concentrations were observed between the groups throughout pregnancy (Table 3). This is particularly interesting given that increased hsCRP levels have been widely reported in conditions such as gestational diabetes and pregnancy hypertensive disorders. Moreover, some studies have proposed hsCRP as a potential biomarker for early screening of pregnancy-related complications [41].

The results of our study have shown that PLTP concentration in the control group is significantly negatively correlated with resistin concentration throughout pregnancy, whereas this association was not observed in the group with complications. To our knowledge, there is no published data on an association between PLTP and resistin in pregnancy. Based on our results, we hypothesize that, in healthy pregnancy, hormonal changes and specific metabolic signals might modulate PLTP and resistin secretion in opposite directions to support the lipid-inflammation interplay essential to maintaining a healthy pregnancy. An increase in resistin concentrations toward the middle of healthy pregnancy reflects a mild inflammatory state, accompanied by impaired HDL structure and function [3]. Opposite-direction changes in PLTP concentrations, as an HDL remodelling factor, could be interpreted as compensatory or feedback mechanisms. This assumption is supported by the absence of this association between PLTP and resistin concentration in pregnancies with complications.

An additional objective of this study was to assess the predictive capacity of *PLTP *gene expression, PLTP concentrations, and other investigated parameters in the first trimester for the onset of cardiometabolic pregnancy complications. Pregestational BMI and *PLTP* gene expression in the first trimester demonstrated significant, independent associations with the development of cardiometabolic pregnancy complications, regardless of other confounders (Table 4). Notably, our findings emphasize the relevance of first-trimester PLTP gene expression as a biomarker that may influence HDL functionality and maternal inflammatory status. These results underscore the need for further investigation into the PLTP role in the pathogenesis and the assessment of risk for cardiometabolic pregnancy complications.

A key limitation of this study is the relatively small sample size, which may have reduced statistical power and affected the robustness of the findings. Furthermore, pregnant women over 35 years of age were not excluded, and the groups were not matched by BMI, both of which represent additional methodological constraints. Another limitation is the inability to assess PLTP activity, which would have provided a more comprehensive evaluation of PLTP function and its potential role in pregnancy physiology and pathology.

## Conclusions

In conclusion, this study provides novel evidence that *PLTP *gene expression and concentration follow similar trajectories throughout trimesters in both healthy and pregnancies with cardiometabolic complications. However, lower *PLTP *gene expression in the first trimester was independently associated with subsequent cardiometabolic pregnancy complications. The observed alterations in advanced lipid parameters (reduced desmosterol HDL concentrations and elevated Cer C24:0 levels), along with increased resistin concentrations, suggest early disturbances in HDL remodelling, lipid metabolism, and inflammatory processes that precede the clinical manifestation of cardiometabolic pregnancy complications. These findings emphasize the importance of early monitoring of lipid and inflammatory pathways during pregnancy.

## Dodatak

### Authors' contribution

M.S., A.Z., J.V., Z.M., and A.S. contributed to the conception and design of the study. M.D., T.A., M.S., M.M., D.A., T.G., S.J., and J.M. were responsible for sample collection, laboratory analysis, and data collection. M.S. and A.S. were responsible for writing the original draft preparation. All authors have read and agreed to the submitted version of the manuscript.

### Funding

This research was supported by the Science Fund of the Republic of Serbia, Grant No. 7741659, High-density lipoprotein MetabolOMe research to improve pregnancy outcome - HI-MOM. The authors appreciate support from the Ministry of Science, Technological Development and Innovation, Republic of Serbia (Grant Agreement with University of Belgrade-Faculty of Pharmacy No: 451-03-65/2024-03/ 200161 and No: 451-03-66/2024-03/ 200161).

### Institutional review board statement

The study complies with the guidelines for human studies and was conducted in accordance with the World Medical Association Declaration of Helsinki. This study was approved by the Ethics Committee of the Gynaecology and Obstetrics Clinic »Narodni front« No: 05006-2020-10738, Ethics Commission of Faculty of Medicine, University of Belgrade, NUMBER: 1322/VII-27, and Ethical Committee for Biomedical Research of the Faculty of Pharmacy, University of Belgrade, No. 1156/2.

### Informed consent statement

Informed consent was obtained from all subjects involved in the study.

### Data availability statement

The data presented in this study are available on request from the corresponding author. The data are not publicly available due to privacy and ethics considerations.

### Conflict of interest statement

All the authors declare that they have no conflict of interest in this work.
